# Variant meso-Rex bypass with transposition of abdominal autogenous vein for the management of idiopathic extrahepatic portal vein obstruction: a retrospective observational study

**DOI:** 10.1186/s12893-015-0101-6

**Published:** 2015-10-17

**Authors:** Tae-Yong Ha, Kyung-Mo Kim, Gi-Young Ko, Seak Hee Oh, Tae-Won Kwon, Yong-Pil Cho, Sung-Gyu Lee

**Affiliations:** Department of Surgery, University of Ulsan College of Medicine and Asan Medical Center, Asanbyeongwon-gil 86, Songpa-gu, Seoul 138-736 Korea; Departments of Pediatrics, University of Ulsan College of Medicine and Asan Medical Center, Asanbyeongwon-gil 86, Songpa-gu, Seoul 138-736 Korea; Departments of Radiology, University of Ulsan College of Medicine and Asan Medical Center, Asanbyeongwon-gil 86, Songpa-gu, Seoul 138-736 Korea

**Keywords:** Portal vein, Obstruction, Meso-Rex bypass

## Abstract

**Background:**

The aim of this study was to evaluate whether variant meso-Rex bypass with transposition of abdominal autogenous vein can be used as an alternative treatment modality for selected patients with symptomatic extrahepatic portal vein obstruction.

**Methods:**

This was a retrospective review of six consecutive patients who received this alternative procedure for the treatment of symptomatic portal hypertension secondary to idiopathic extrahepatic portal vein obstruction. Their clinical characteristics, operative procedures and outcomes were analyzed retrospectively.

**Results:**

The procedure was attempted in six patients, and all had a patent shunt established by intraoperative portography at the end of the procedure; the coronary vein was used in four patients and the inferior mesenteric vein was used in two. During the median period of 23.5 months (range 10–30 months), follow-up was uneventful except one patient; reduced portal hypertension and no new episodes of gastrointestinal bleeding were observed in all patients, with the exception of one patient with shunt stenosis and recurrent varix bleeding who had to undergo endovascular treatment to restore portal vein blood flow. Technical and clinical success was achieved in all patients.

**Conclusions:**

This procedure could be used safely and effectively to treat selected patients with portal hypertension secondary to extrahepatic portal vein obstruction.

## Background

For the management of idiopathic extrahepatic portal vein obstruction (EHPVO), the meso-Rex bypass (MRB) restores portal inflow to the liver by inserting a venous conduit between a splanchnic vein and the intrahepatic left portal vein (PV) branch in the Rex fossa. This procedure was initially indicated for the treatment of extrahepatic PV thrombosis following liver transplantation in children, but has been successfully used to treat non-transplant patients with thrombosis caused by other etiologies [1–5]. The standard MRB technique uses the internal jugular vein graft of the patient to restore hepatopetal flow, and results in satisfactory long-term patency and a significantly reduced rate of clinical complications [1–5]. However, inevitably, this procedure requires neck exploration and sacrifice of the internal jugular vein, and some reports have mentioned problems secondary to the procurement of the internal jugular vein [5, 6].

Previously, we described a method involving the transposition of a coronary vein, which is enlarged in most cases of portal hypertension, as an alternative to the standard MRB technique [7]. The aim of this study was to evaluate whether variant MRB with transposition of abdominal autogenous vein is a safe and effective treatment modality in selected patients with symptomatic EHPVO who are refractory to conservative management.

## Methods

This observational study included six consecutive patients who presented with symptomatic portal hypertension secondary to idiopathic EHPVO and underwent variant MRB with transposition of abdominal autogenous vein at our hospital. The study protocol (Asan Medical Center IRB No. 2013–1068) was approved by the hospital Institutional Review Board, and all the guardians and two (Patients 1 and 3) of the patients provided written informed consent. Consent to publish all of the information provided in Table 1 was also obtained from all the participants.

**Table 1:** Patient characteristics

		Combined anomaly	Clinical symptoms	Preoperative Duplex USG		Embolization of portosystemic shunt	Combined splenectomy	Follow-up (months)	
Patient	Sex/age (yr)				Graft type				Outcome
1	M/20	-	EV, S, HS	Absent	CV	+	+	30	Good evolution
2^a^	M/1	+	EV, S	Present	CV	+	-	30	Good evolution
3	M/19	-	EV, S, HS	Absent	CV	+	-	24	Good evolution
4	M/10	-	EV, S, HS	Present	IMV	+	-	23	Good evolution
5	F/4	+	EV, S, HS	Absent	IMV	-	-	10	Good evolution
6^b^	M/13	+	EV, S, HS	Absent	CV	-	+	10	Good evolution

Combined anomalies: Patient 2, dextrocardia and intestinal malrotation; Patients 5, congenital heart disease; Patient 6, imperforate anus

Preoperative Duplex USG: Present, visualization of intrahepatic portal vein; Absent, non-visualization of intrahepatic portal vein

*EV* esophageal varix bleeding, *S* splenomegaly, *HS* hypersplenism, *USG* ultrasonography, *CV* coronary vein, *IMV* inferior mesenteric vein

^a^Percutaneous transluminal angioplasty at 19 months after meso-Rex bypass

^b^Past history of distal spleno-renal shunt operation at 58 months before meso-Rex bypass

The male-to-female ratio was 5:1, and the median age at surgery was 11.5 years (range 1–20 years). All patients had a history of upper gastrointestinal bleeding, splenomegaly, severe hypersplenism, or some combination thereof. Both Doppler ultrasonography and computed tomography (CT) angiography were done to assess the intrahepatic PV. A preoperative percutaneous transhepatic liver needle biopsy was performed under general anesthesia to exclude significant parenchymal hepatic fibrosis.

The surgical technique used was described previously [7]. If the coronary vein had an adequate diameter and flow in the hepatopetal direction, this vein was fully mobilized, divided, and then transposed to the ventral portion of the intrahepatic left PV to which it was anastomosed end-to-side using non-absorbable monofilament interrupted sutures. If there was no adequate coronary vein, the inferior mesenteric vein was used. Intraoperative portography confirmed adequate portal blood flow into the liver, and the remaining large portosystemic shunts were embolized with coils to augment portal blood flow to the liver, if required. Combined splenectomy was performed in patients with massive splenomegaly.

After the operation, patients were anticoagulated with a regimen of low molecular weight heparin for 14 days followed by orally administered warfarin for 3 to 6 months. All patients were routinely followed using Doppler ultrasonography to assess shunt patency on day 0, 1, and 4, and CT angiography on day 5. After discharge, Doppler ultrasonography was performed to assess shunt patency at 1, 3, 6, and 12 months. Routine blood and biochemical parameters were also evaluated at 1, 3, 6, and 12 months, and each year thereafter.

Success of the variant MRB was defined as shunt patency at 6-month follow-up, as assessed by Doppler ultrasonography with visualization of the shunt without stenosis [4]. If a patient had a repeat intervention that was successful in dilating a stenosed or thrombosed shunt, the variant MRB procedure was considered a success. On the other hand, if repeated attempts at reperfusion of a stenosed or thrombosed shunt were unsuccessful, the procedure was considered a failure.

## Results and discussion

**Fig. 1:**
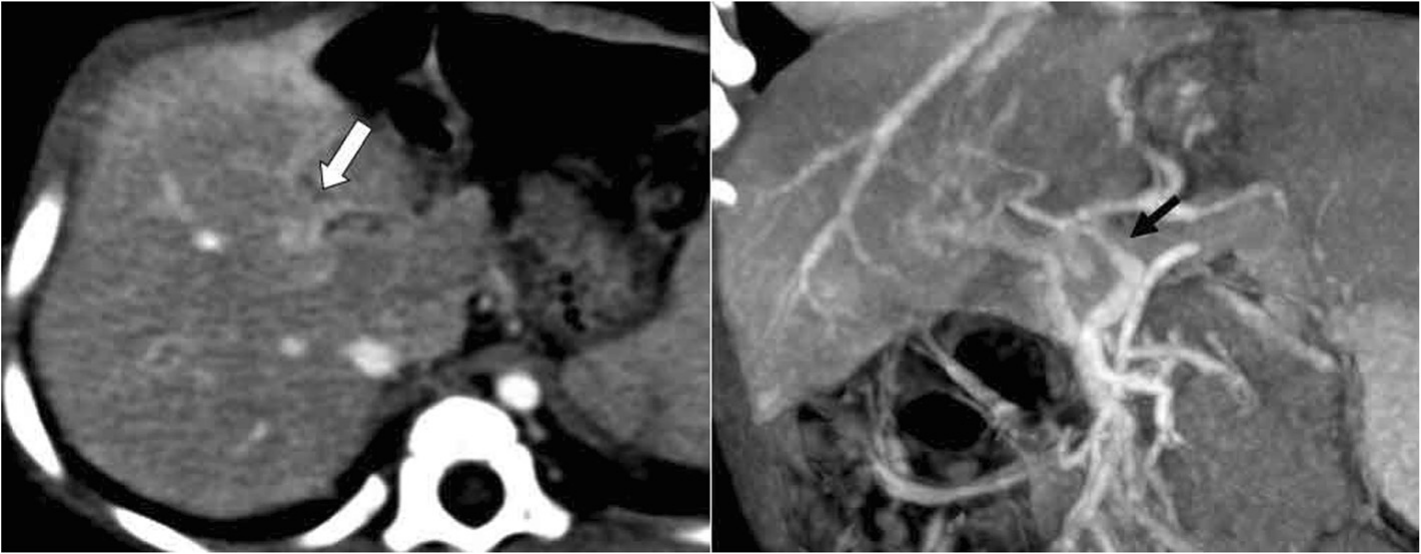
Preoperative computed tomographic angiography. Illustration: Preoperative computed tomographic angiography images showing an obliterated main (*black arrow*) and left (*white arrow*) intrahepatic portal vein

The characteristics of the patients are summarized in Table 1. Six consecutive patients showed symptomatic portal hypertension secondary to idiopathic EHPVO during the study period. None of them had a familial history of thrombosis or neonatal history of umbilical vein catheterization. Combined anomalies were noted in three patients. The first clinical manifestation was varix bleeding in five patients and hypersplenism in one patient at a median age of 24 months (range 10 months–14 years). Preoperative Doppler ultrasonography and CT angiography revealed thrombosis or cavernous transformation of the main PV in all patients (Fig. 1); two patients had an intrahepatic PV that was adequate for shunting, but blood flow in the left PV in the other four patients was inadequate. In these four patients, patency was verified by surgical exploration.

**Fig. 2:**
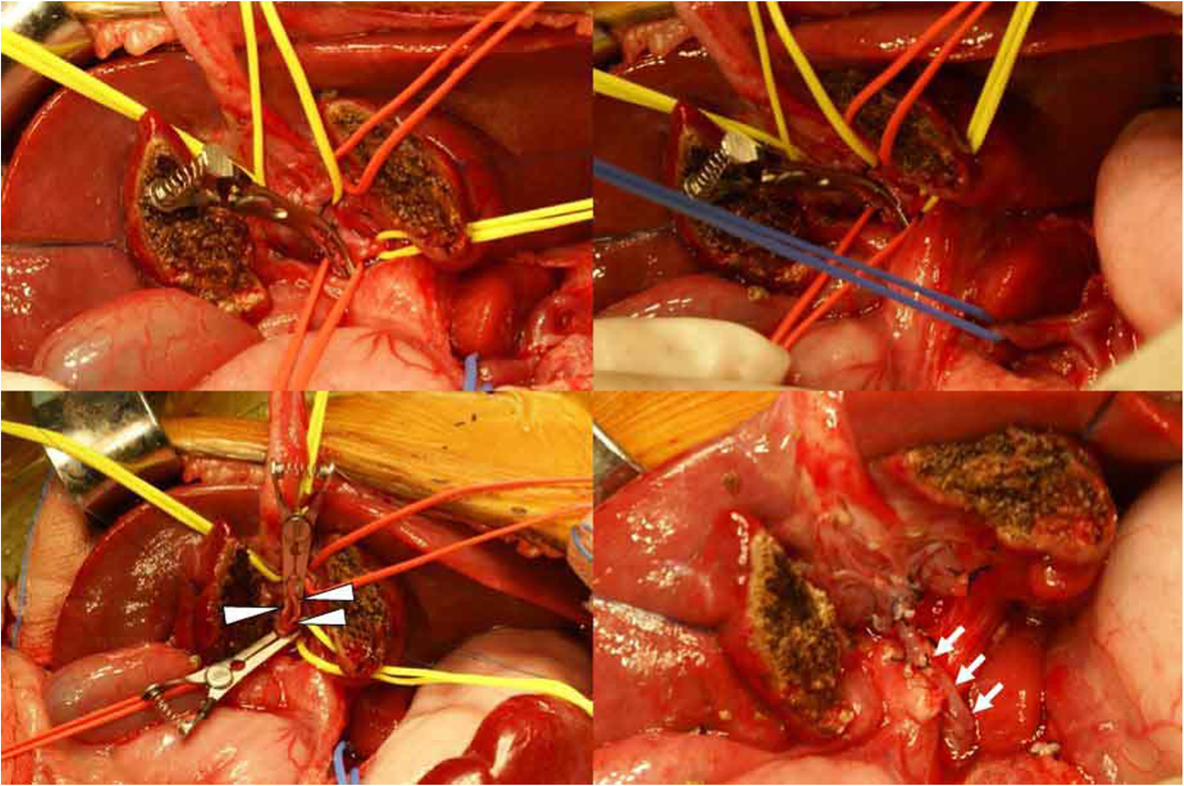
Operative findings. Illustration: Operative findings showing the transposed coronary vein (*white arrows*) anastomosed end-to-side to the ventral portion of the extrahepatic left portal vein (*white arrowheads*) using non-absorbable monofilament interrupted sutures

**Fig. 3:**
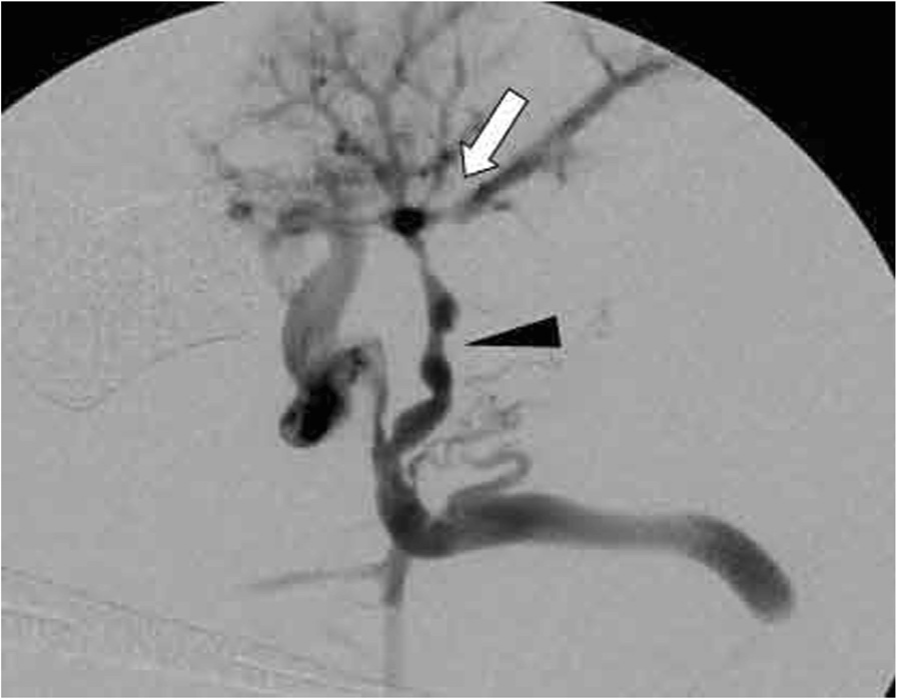
Intraoperative portography. Illustration: Intraoperative portography at the end of the procedure showing a brisk flow to the left intrahepatic portal vein (*white arrow*) via the variant meso-Rex bypass (*black arrowhead*)

**Fig. 4:**
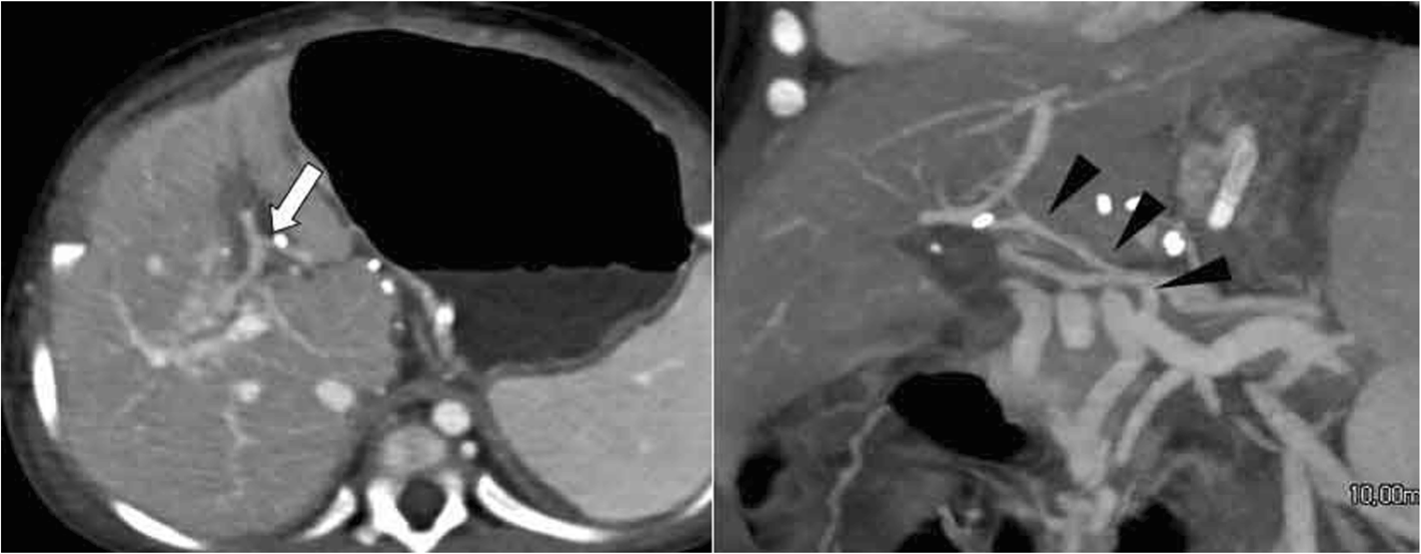
Follow-up computed tomographic angiography. Illustration: Follow-up computed tomographic angiography images showing increased portal flow (*white arrow*) via the preserved variant meso-Rex bypass (*black arrowheads*)

A variant MRB with transposition of abdominal autogenous vein was attempted in six patients (Fig. 2), and all patients had a patent shunt established by intraoperative portography (Fig. 3); the coronary vein was used in four patients and the inferior mesenteric vein was used in two. Intraoperative embolization of the remaining portosystemic shunts was done with coils to augment portal blood flow to the liver in four patients, and combined splenectomy was performed in two patients because of massive splenomegaly. Postoperative recovery was rapid and uneventful in all patients. The results of liver function tests were within normal ranges and normal portal blood flow was observed by Doppler ultrasonography and CT angiography (Fig. 4). There was no operation-related morbidity or mortality.

Long-term patency was achieved in all patients as confirmed by follow-up Doppler ultrasonography and CT angiography. During the median period of 23.5 months, follow-up was uneventful except one patient; portal hypertension showed signs of improvement and no new episodes of gastrointestinal bleeding occurred. There was one exception; one patient showed recurrent varix bleeding (Patient 2) at 19 months after variant MRB and shunt stenosis on Doppler ultrasonography. This patient received endovascular treatment, which was successful in restoring portal blood flow. In this study, of the six patients who underwent a variant MRB with transposition of abdominal autogenous vein, technical and clinical success was achieved in all patients. There was no long-term operation-related complication.

The incidence and natural history of EHPVO have not been completely characterized; however, morbidity has been mainly linked to symptoms of portal hypertension [3–8]. Despite conservative treatment, patients who persistently present with clinically significant symptoms of portal hypertension are directed toward surgery options, including MRB, which is the treatment of choice to restore physiologic PV blood flow to the liver [3–11]. This technique not only effectively resolves or prevents all of the known complications of EHPVO, but also shows metabolic benefits over various portosystemic shunt operations [3–8].

For successful application of the MRB, eligible patients must fulfill two preconditions; the hepatic structure must be within normal limits and the umbilical portion of the left PV must stay patent. In patients with EHPVO, the initial thrombotic process seems to mainly involve the PV trunk, to which it is limited under typical disease conditions; a variable extension, either downstream into the intrahepatic radicals, or upstream into the splanchnic system, or both can be observed [5]. Although extension of thrombosis to the intrahepatic left PV sometimes precludes the use of a MRB, this technique is surgically feasible in most patients without acute liver necrosis or liver fibrosis, even when the intrahepatic left PV is poorly visible or not seen at all on routine preoperative imaging studies [7, 8]. In such cases, the size and patency of the umbilical portion of the left PV must be confirmed by surgical exploration. Although a preoperative Doppler ultrasonography evaluation did not indicate blood flow in the intrahepatic left PV in four patients in our study, surgical exploration revealed that the intrahepatic PVs were hypoplastic but appropriate for shunting.

In the standard MRB technique consisting of bypass of the thrombosed portal trunk via the interposition of a graft between the superior mesenteric vein and the Rex recessus, the patient’s internal jugular vein is the most commonly used source of the vascular autograft, and the implementation of this procedure has yielded excellent results over the past two decades [9–13]. However, inevitably, this procedure requires neck exploration and sacrifice of the internal jugular vein, and some reports have mentioned problems secondary to the procurement of the internal jugular vein [5, 6].

The current technique, using a transposed abdominal autogenous vein as a conduit, without neck exploration, is a potentially valuable modification of conventional MRB in selected patients with an enlarged coronary or a patent inferior mesenteric vein [14, 15]. It would be valuable in relieving symptoms of portal hypertension and hypersplenism in non-transplant patients with EHPVO. Furthermore, this technique would simplify the operative procedure as it involves only one vascular anastomosis; thus, it decreases the total operating time, eliminates the procurement of autologous veins, and reduces the need for simultaneous embolization of large collaterals to augment portal blood flow.

Several limitations should be noted. The retrospective nature of the analysis, the small sample size, and the short follow-up duration make it particularly challenging to reach definitive conclusions about the safety and efficacy of this alternative technique. Since these data were collected from a single institution, our results cannot be generalized to other centers. Furthermore, the decision to perform a variant MRB was made by the surgeon based on the expected level of the adequacy of an enlarged coronary or a patent inferior mesenteric vein. To the best of our knowledge, there is no general consensus regarding optimal criteria of a suitable autogenous venous conduit for successful standard MRB or our current technique, because of its rarity. Future prospective studies on larger cohorts are warranted.

## Conclusions

In the present small series, we propose an alternative technique for the transposition of abdominal autogenous vein for the management of EHPVO without a vascular conduit, which simplifies the operative procedures. This technique could be used safely and effectively to treat symptoms of portal hypertension in most cases of EHPVO with an enlarged coronary or a patent inferior mesenteric vein.

## Abbreviations

CT: Computed tomography
EHPVO: Extrahepatic portal vein obstruction
MRB: Meso-Rex bypass
PV: Portal vein
